# The Invasive Round Goby *Neogobius melanostomus* as a Potential Threat to Native Crayfish Populations

**DOI:** 10.3390/ani11082377

**Published:** 2021-08-12

**Authors:** Pavel Franta, Radek Gebauer, Lukáš Veselý, Miloš Buřič, Natalia Z. Szydłowska, Bořek Drozd

**Affiliations:** South Bohemian Research Center of Aquaculture and Biodiversity of Hydrocenoses, Faculty of Fisheries and Protection of Waters, University of South Bohemia in České Budějovice, Zátiší 728/II, 389 25 Vodňany, Czech Republic; rgebauer@frov.jcu.cz (R.G.); veselyl@frov.jcu.cz (L.V.); buric@frov.jcu.cz (M.B.); nszydlowska@frov.jcu.cz (N.Z.S.); drozd@frov.jcu.cz (B.D.)

**Keywords:** *Asellus aquaticus*, biological invasion, ecological impact, prey preferences, functional response, *Procambarus virginalis*

## Abstract

**Simple Summary:**

*Neogobius melanostomus* is a highly invasive fish that has colonized most major European rivers and is dispersing into their tributaries. Its foraging behaviour does not show particular prey preferences, which makes predicting its interactions with endangered members of the macrozoobenthic community in tributaries a challenge. We observed the interaction of *N. melanostomus* and crayfish juvenile or *A. aquaticus* in single- and multiple-prey systems to better predict its ecological impact. The results suggest an impact of *N. melanostomus* on crayfish similar to that on *A. aquaticus*, potentially making it a threat to crayfish population stability. Destabilization of a keystone species such as crayfish in river tributaries may lead to a trophic cascade in the ecosystem with irreversible consequences.

**Abstract:**

Despite the spread of round goby *Neogobius melanostomus* into freshwater streams, there is a lack of information with respect to its effect on macroinvertebrate communities, especially crustaceans. We studied foraging efficiency of *N. melanostomus* on *Procambarus virginalis* and *Asellus aquaticus*, using a functional response (FR) approach. Stocking density of the prey species was manipulated to determine its effect on consumer utilization, with prey offered separately or combined at 1:1, 3:1, and 1:3 at each tested density. For both prey species, *N. melanostomus* exhibited type II FR, occasionally with a high proportion of non-consumptive mortality. *Procambarus virginalis* suffered a significantly higher attack rate compared to *A. aquaticus*. *Neogobius melanostomus* killed significantly more of the most prevalent prey, regardless of species. In trials with prey species of equal proportions, a difference in the number of each species killed was observed only at the highest density, at which *P. virginalis* was preferred. *Neogobius melanostomus* may be an important driver of population dynamics of prey species in the wild. The non-selective prey consumption makes *N. melanostomus* a potential threat to macrozoobenthic communities of river tributaries.

## 1. Introduction

Crayfish have an impact at multiple trophic levels through predation, shedding, burrowing, and competition [1,2,3] and are considered keystone species influencing stability and functionality of ecosystems, particularly in tributaries to major streams [4,5,6]. Crayfish populations worldwide are threatened by multiple stressors: Climate change, water pollution, habitat modification, invasive species, and disease [5,7]. Nearly one third of crayfish species worldwide are threatened with extinction [7]. Although interventions in the EU [8] and throughout the world [9,10] aim to improve the ecological status of freshwater lotic ecosystems, the threat presented by non-indigenous species is ever-increasing [11]. In addition to interactions with non-indigenous crayfish, native crayfish interact with small benthic fishes, including non-native species [1].

The round goby *Neogobius melanostomus* (Pallas 1814), among the most invasive of freshwater fish species [12], has expanded substantially beyond its native range the Ponto-Caspian region. It poses a serious threat to freshwater and brackish ecosystems [13] causing critical food web disruptions, shifts in trophic levels, extermination of native species through direct predation and/or competition for resources and habitat, and spread of disease [14,15,16,17]. In major rivers, after establishing a viable population, *N. melanostomus* spreads both down- and up-stream [18,19]. It is increasingly found in tributaries of major rivers [20,21,22] that are often used as refugia for native species [23] and contain unique highly diverse macrozoobenthic communities including endangered species such as crayfish [24]. These communities may be seriously threated by *N. melanostomus* invasion and dispersion [25,26].

Macrozoobenthos represent a predominant proportion of the *N. melanostomus* diet [27,28], reflecting the community structure in a given locality [29,30]. In contrast to major rivers and lakes, which often harbour several non-native macrozoobenthos species, in small streams with highly diverse macrozoobenthic communities, *N. melanostomus* remains a generalist omnivore [30]. This can lead to a significant transformation of the community structure with severe consequences to endangered species, since even partial depletion of a single prey population can alter the predator food selectivity [31]. Nevertheless, crayfish are rarely reported in *N. melanostomus* diet in invaded regions [32,33], possibly the result of a unique flip-tail escape strategy, as observed for dragonfly nymph predation on early-stage crayfish [34].

With respect to the coexistence of small benthic fish and crayfish, due to similar body size, the primary focus has been on competition for food and shelter and on behaviour interactions in the presence of a common predator, as opposed to their mutual predation relationship [1]. However, crayfish juveniles that have become independent after leaving the female are threatened by fish predation due to their small size [35,36] and limited antipredator defences, usually restricted to the tail-flip escape movement [35,36,37,38]. The impact of small voracious benthic fish such as *N. melanostomus* on early crayfish stages may be intensified when sharing a common habitat. The ecological impact of *N. melanostomus* on crayfish populations has not been quantified.

Understanding and predicting novel predator-prey interaction dynamics and their consequences for invaded freshwater communities is a critical issue in invasion management [39]. Invasive predators, often possessing better foraging efficiency and/or resource utilization, may have higher maximum feeding rates than the analogous native predators and therefore greater ecological impact [40,41] with especially pronounced consequences in aquatic environments [42].

Resource availability represents a crucial determinant of feeding rate as illustrated by a functional response (FR) curve [43,44]. The shape and asymptote of the curve depict important parameters of consumer-resource interactions and population community dynamics [45,46]. Invasive species often display elevated FRs compared to native or low-impact non-native ecologically analogous species [47,48,49] making comparative FR a valuable tool for invasion biologists [48,49,50]. Functional response has been calculated for comparison of *N. melanostomus* foraging efficiency with native [51] as well as non-native analogous species [52] and can be employed for comparison of predator impact on prey components, since predator response to prey may be prey species–dependent [53,54,55,56,57]. A higher FR asymptote denotes more effective prey exploitation, possibly due to greater prey attractiveness or palatability and/or greater predator adaptation to prey antipredation behaviour. Currently, knowledge of the relationship between *N. melanostomus* and crayfishes is lacking, especially in tributaries serving as refuges for native aquatic biota and sources of genetic diversity for main stream ecosystems.

The aim of our study was to characterize *N. melanostomus* foraging efficiency on early juvenile crayfish. While natural ecosystems generally consist of multiple prey species per predator, the majority of research experiments address interaction between a single predator and prey species. We observed the predation behaviour of *N. melanostomus* in the presence of two prey species differing in escape behaviour at several densities and stocking proportions. We hypothesized that prey defence, as well as the presence of an alternative prey in various proportions, may significantly influence predator foraging efficiency.

## 2. Materials and Methods

### 2.1. Predator and Prey Acquisition and Acclimatization

*Neogobius melanostomus* were collected with a backpack pulsed-DC electrofishing unit (FEG 1500, EFKO, Leutkirch, Germany) in early October 2018 from a recently colonized locality in the Elbe River (50.6524583 N, 14.0441314 E). Specimens (TL = 55.9 ± 2.6 mm; W = 2.1 ± 0.3 g) were transported to the Institute of Aquaculture and Protection of Water and acclimated in a 1600 L recirculating aquaculture system for 7 days. They were fed frozen chironomid larvae to satiation twice daily. Water temperature (20.3 ± 0.3 °C), dissolved oxygen (100.6 ± 2.9%), and pH (7.7 ± 0.2) were measured twice daily with an HQ40d digital multimeter (Hach Lange GmbH, Düsseldorf, Germany).

We used two hard-bodied benthic invertebrate prey species of similar body mass differing in escape strategy: The native water louse *Asellus aquaticus* (L.) (W = 5.56 ± 1.94 mg) is representative of isopods that form a component of the *N. melanostomus* diet [58,59]. Isopod locomotion is restricted to slow crawling with no escape strategy [60]. The second species was the juvenile non-native marbled crayfish *Procambarus virginalis* (Lyko 2017) (W = 5.45 ± 0.66 mg), a common crayfish model species for laboratory research [61], which exhibits a flip-tail escape strategy as the native crayfish species [34]. Both native crayfish species in the Czech Republic (i.e., *Astacus astacus* and *Austropotamobius torretium*) are classified as critically endangered species in the Red list of threatened species of the Czech Republic with a continual populations decline [62]. Therefore, their use for experiments performance is strongly forbidden and dispensation from law is impossible. 

*Asellus aquaticus* was collected with hand nets in late September 2018 in the Kyselá voda stream (49.0195475 N, 14.4640344 E). The *P. virginalis* were obtained from the Laboratory of Ethology of Fish and Crayfish, FFPW USB. Both prey species were housed in 200 L glass aquaria equipped with PVC trickling filter media (Hewitech GmbH, Ochtrup, Germany) that served as shelter and filter. Half the water volume was exchanged daily with dechlorinated tap water.

### 2.2. Experiment Design

Transparent plastic boxes (295 × 185 × 155 mm; total volume = 6000 mL) filled with 5000 mL dechlorinated tap water and 200 mL fine aquarium sand (particle size < 0.3 mm) were used as experimental arenas. Five prey exposures were tested: *A. aquaticus* and *P. virginalis* separately and combined at respective ratios of 1:1, 1:3, and 3:1. Each exposure included prey densities of 4, 8, 20, 36, 60, and 100 individuals/box with six replicates per density. Overall, 180 *N. melanostomus* specimens were used in the experiment, whereas each predator was used only once. Baseline prey mortality was assessed with control groups of the same combinations, ratios, and densities in six replications without predators. *Neogobius melanostomus* were starved for 24 h before each trial to standardize hunger level and placed individually into the experimental arenas 1 h after prey insertion. A light regime of 500 lux m^2^ was maintained in a 12 L:12 D photoperiod. The predator was removed from the arena after 24 h, and the number and species of surviving prey and non-consumptive mortality (NCM) were determined. Non-consumptive mortality was calculated as in [63] including dead prey not ingested by the predator. Each predator was used once to avoid experience bias. 

### 2.3. Data Analysis

The FR of *N. melanostomus* was fitted separately for each prey organism and ratio and calculated as a total number of killed prey (sum of NCM and eaten prey). Hence, FR quantified the overall impact of *N. melanostomus* on prey. The FRs of *N. melanostomus* on prey were compared between species and among stocking ratios. The type of FR was determined by fitting of logistic regression on the basis of the relationship between the killed prey (*N_e_*) and the initial prey density (*N*_0_):

(1) $$\frac{N_{e}}{N_{0}}=\frac{\exp\left(P_{0}+P_{1}N_{0}+P_{2}N_{0}^{2}+P_{3}N_{0}^{3}\right)}{1+\exp\left(P_{0}+P_{1}N_{0}+P_{2}N_{0}^{2}+P_{3}N_{0}^{3}\right)}$$

where *P*_0_, *P*_1_, *P*_2_, and *P*_3_ represent intercept, linear, quadratic, and cubic coefficients, respectively, estimated using the method of maximum likelihood. If *P*_1_ reaches a positive value with *P*_2_ negative, the proportion of prey killed is positively density-dependent, which is peculiar to type III FR. However, if *P*_1_ is a negative value, the proportion of prey killed declines monotonically from initial prey density, indicating type II FR [46]. Based on logistic regression, we used Rogers’s random predator equation [64] for type II FR in all prey types and ratios, which is suitable for non-replacement design:

(2) $$N_{e}=N_{0}-\left(1-\exp\left(a\left(N_{e}h-T\right)\right)\right)$$

where *T* is time of prey exposure to predator (24 h), a is predator attack rate (predator relative consumption rate corresponds to search efficiency in low prey density manifested in an initial slope steepness on FR curve; L day-1), and h is predator handling time (time pursuing, subduing, and eating of prey combined with time spent prey searching and digestive pause; days prey-1) [65]. For bordering of the Rogers’s random-predator equation by Ne on both sides of the equation, we used the Lambert W function for solving Equation [66]:

(3) $$N_{e}=N_{0}-\frac{W\left\{ahN_{0\;}\;exp\left[-a\left(T-hN_{0}\right)\right]\right\}}{ah}$$

We estimated parameters a and h using non-linear least-squares regression and Lamber *W* function included in the EMDBOOK package [66]. Differences in parameters among prey species and ratios were evaluated based on an overlap of 95% confidence intervals. If no overlap was observed, the parameters significantly differed among the treatments [67].

The effects of prey species, ratio, density, and their interaction upon the number of prey eaten, NCM, and killed prey were tested using a generalized linear model (GLM) with Gaussian distribution. Tukey’s HSD post-hoc test was subsequently used for determination of significant differences among exposures. Since the survival rate in all control treatments exceeded 97% (97.2–100.0%), the mortality of predator-exposed prey was attributed exclusively to the presence of *N. melanostomus*, and datasets were not adjusted for natural mortality. All analyses were conducted in R version 4.0.3 (R Development Core Team 2018). 

## 3. Results

### 3.1. Functional Response Type

**Figure 1:**
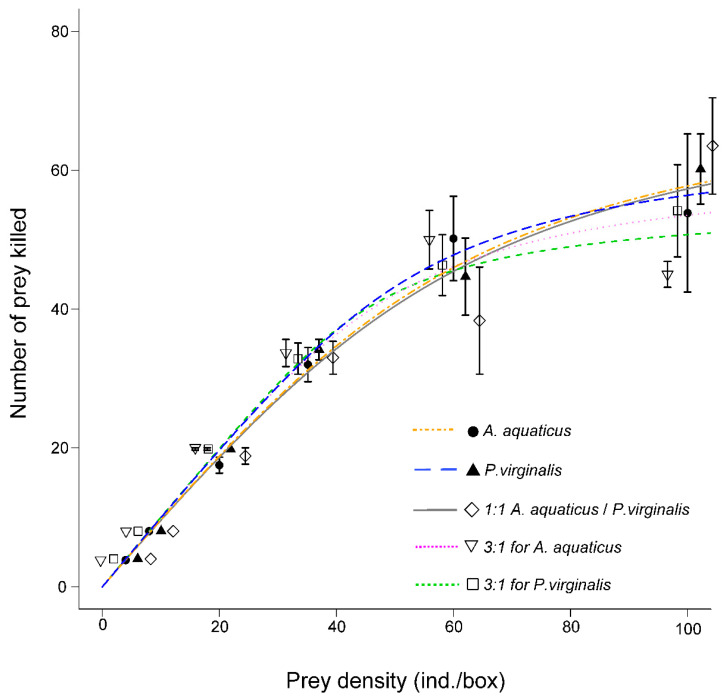
Functional response (mean ± SE) of *Neogobius melanostomus*. *Asellus aquaticus* is represented by the orange dot-dash line and *Procambarus virginalis* by the blue dashed line. Prey were offered separately and combined 1:1 (grey solid line), 3:1 for *A. aquaticus* (pink dotted line), and 3:1 for *P. virginalis* (green dotted line).

In all exposures, *N. melanostomus* exhibited the type II functional response (Figure 1): Significant negative linear coefficients in logistic regressions (Table 1).

### 3.2. Attack Rate and Handling Time

**Figure 2:**
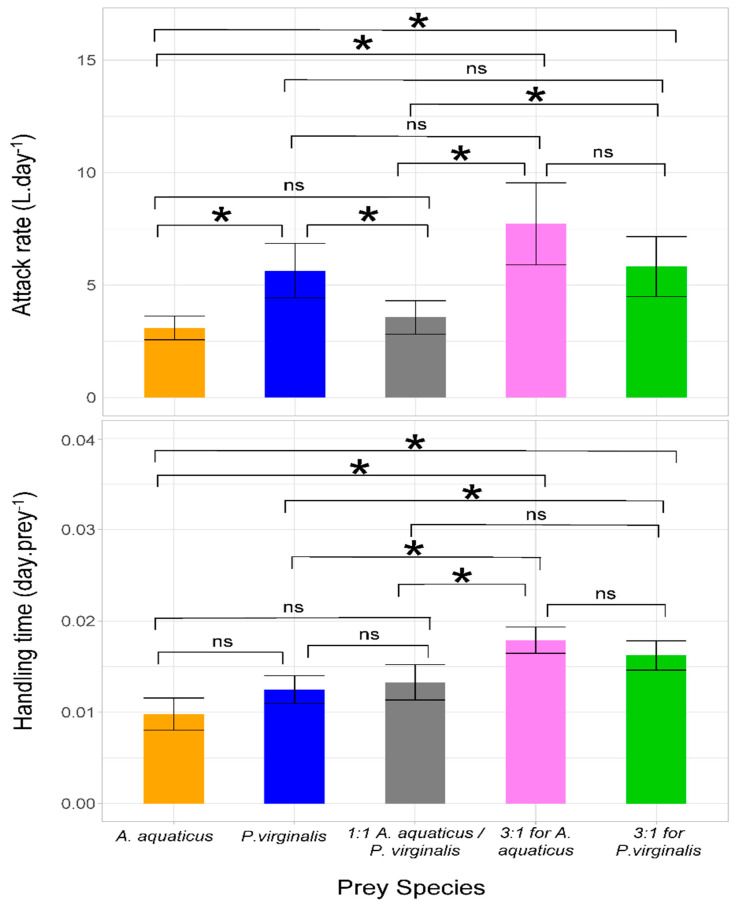
Attack rate and handling time (error bars denote 95% confidence intervals) of *Neogobius melanostomus* with respect to prey species separately and combined. In multiple prey trials, *Asellus aquaticus* and *Procambarus virginalis* were offered at ratios of 1:1, 3:1, and 1:3. Asterisks denote significant (*p* < 0.05) differences among trials and NS indicates non-significant difference.

Significantly higher values of attack rate were observed in the trial with *P. virginalis* offered separately as well as in both 3:1 prey combinations compared with the 1:1 combination and *A. aquaticus* offered separately. *Neogobius melanostomus* displayed the highest handling time in the 3:1 trials, with no significant differences among groups in which prey species were offered separately or at 1:1 (Table 2 and Figure 2).

### 3.3. Number of Killed and Eaten Prey and Non-Consumptive Mortality

**Figure 3:**
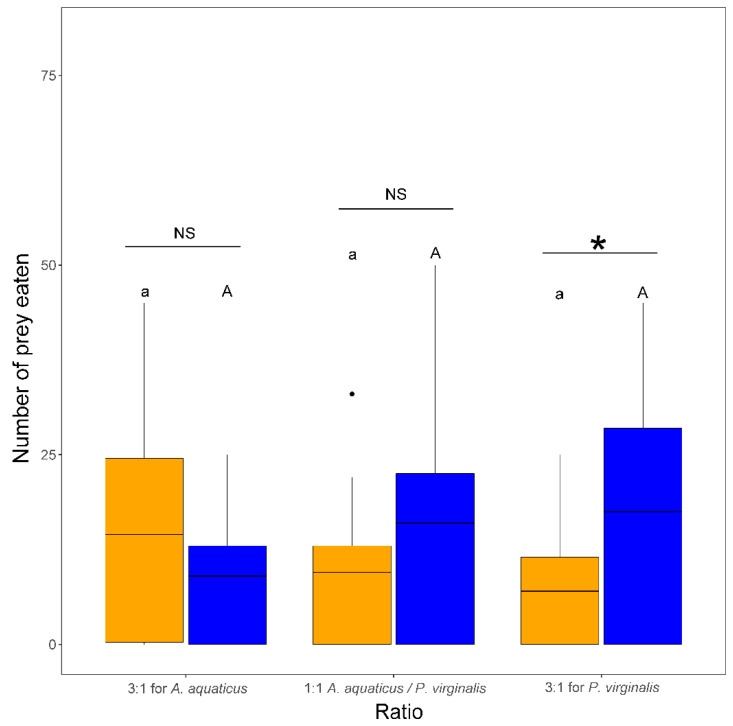
Number of *Asellus aquaticus* (orange) vs. *Procambarus virginalis* (blue) consumed by *Neogobius melanostomus* is prey species ratio–dependent. Exposures with the same letter do not significantly differ (*p* > 0.05). Asterisk denotes significant difference (*p* < 0.05) between species and NS indicates non-significant difference. The points denote outliers.

The number of prey eaten by *N. melanostomus* was significantly affected by the interaction of species and ratio (F_2,102_ = 4.71, *p* = 0.011). This was reflected in a significantly higher number of *P. virginalis* consumed than *A. aquaticus* in the group with 3:1 for *P. virginalis*. There were no other significant differences among trials in the number of prey eaten (Figure 3). 

**Figure 4:**
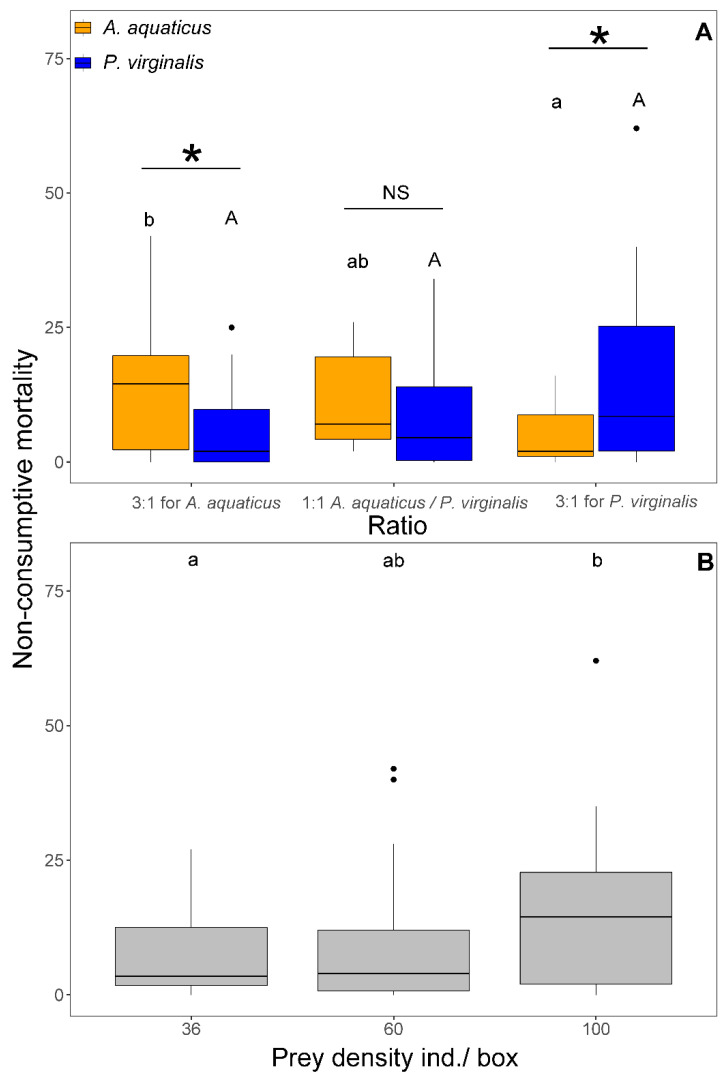
Number of non-consumptive mortality of *Asellus aquaticus* (orange) and *Procambarus virginalis* (blue) by *Neogobius melanostomus* relative to the prey species ratio (**A**) and density (**B**). Effect of density, regardless of prey species, is shown for 1:1 (grey colour) at the highest prey densities (36, 60, and 100 individuals/box). Treatments with the same letter did not differ significantly (*p* > 0.05). Asterisk indicates significant difference (*p* < 0.05) and NS indicates non-significant difference. The points denote outliers.

The NCM was affected by prey density (F_1,103_ = 7.33, *p* = 0.008) and the interaction between prey species and ratio (F_2,101_ = 5.87, *p* = 0.004). The NCM at 1:1 was significantly higher at the highest density (100 ind/box) than at densities < 60 ind/box at the same ratio (Figure 4B). There was no difference in NCM of *A. aquaticus* among the three ratios. In contrast, the NCM of *P. virginalis* at 3:1 for *P. virginalis* was significantly higher than 3:1 for *A. aquaticus*. The NCM was always significantly higher in the prevalent prey species than in the less abundant (Figure 4A). At 1:1, no significant species differences were observed in NCM (Figure 4B). In all exposures, NCM ranged from 0 to 100% of killed prey.

**Figure 5:**
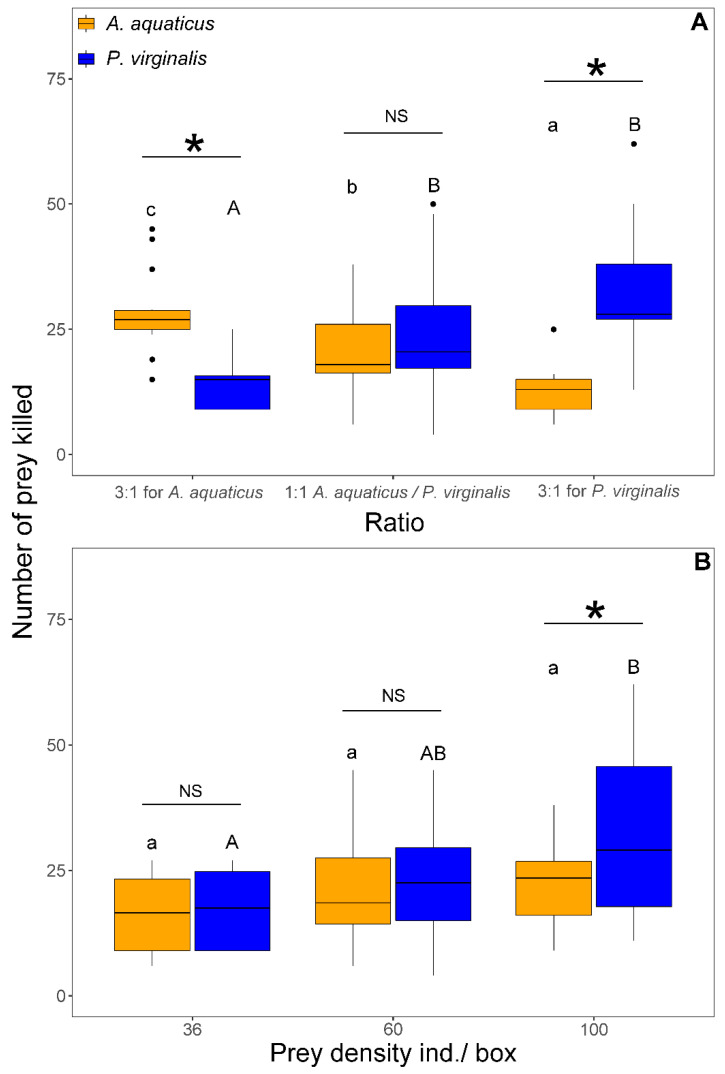
Number of killed *Asellus aquaticus* (orange) and *Procambarus virginalis* (blue) by *Neogobius melanostomus* relative to prey species ratio (**A**) and density (**B**). Effect of density is shown only for 1:1 at 36, 60, and 100 individuals/box. Exposures with the same letter did not significantly differ (*p* > 0.05). Asterisk indicates significant difference (*p* < 0.05) between groups and NS indicates non-significant difference. The points denote outliers.

The number of killed prey was significantly affected by prey density (F_1,103_ = 29.82, *p* < 0.001), species (F_1,106_ = 4.21, *p* = 0.042), and interaction of prey species with prey ratio (F_2,101_ = 40.07, *p* < 0.001) and density (F_1,100_ = 6.13, *p* = 0.015). In both 3:1 trials, *N. melanostomus* killed a significantly higher number of the prevalent prey species. The number of killed *A. aquaticus* differed significantly with the proportion and reflected the number offered. In contrast, the number of killed *P. virginalis* reached similar values at 1:1 and 3:1 for *P. virginalis* only at 3:1 for *A. aquaticus* and was significantly lower than at other ratios (Figure 5A). At 1:1, there was no significant difference between species in the number of killed prey at densities <60 individuals/box. With 100 individuals/box at 1:1, *N. melanostomus* killed significantly more *P. virginalis* than *A. aquaticus* (Figure 5B).

## 4. Discussion

The ability to utilize different prey sources and to switch among prey species as required is an attribute of successful invasive predators that can negatively affect not only prey species populations but also coenoses stability [31,68]. *Neogobius melanostomus* significantly changes composition of the macrozoobenthic communities in the invaded freshwater ecosystems [25,69]. Tributaries of major rivers serve as refuges for native aquatic biota and as sources of genetic diversity for the main streams [23] that are currently heavily affected by biological invasions [70].

*Neogobius melanostomus* exhibited type II FR toward prey organisms differing in escape strategy regardless of presentation. This type of functional response is typical of carnivorous predators [63,71] and is usually associated with destabilization of prey organism populations [72]. Type II FR was previously observed in *N. melanostomus* towards amphipods [49,51,73], *A. aquaticus* [49], and common carp *Cyprinus carpio* L. larvae [52] under experimental conditions. With increasing habitat complexity [74], switching among prey types [45] and consumption of less preferred prey [75] or prey with a well-developed antipredator defence [76] commonly involves a shift from type II FR to type III FR. However, this expected phenomenon was not observed in our two prey–species system, although prey organisms displayed different escape abilities. This is consistent with Gebauer et al. [77] who found no shift in *N. melanostomus* FR with increased habitat complexity, suggesting that *N. melanostomus* is a highly effective predator irrespective of habitat conditions [76] and prey behaviour (this study). 

Handling time, as the ability to find and process prey, determines the predator maximum feeding rate [50]. This parameter closely correlates with habitat complexity [57,77,78] and, especially, with prey morphology and behaviour [53,79]. The typical crayfish flip-tail escape is generally considered a successful antipredation strategy [34,80] that reduces predator success or at least requires higher predator energy [81,82]. Contrary to expectations, we observed no significant differences in handling time of *A. aquaticus* and *P. virginalis*, suggesting that the crayfish escape strategy is ineffective against *N. melanostomus* predation, at least in early crayfish ontogenetic stages and in sandy substrates. In the trials with a single prey species at low density, *N. melanostomus* exploited *P. virginalis* more effectively than *A. aquaticus*, reflected in its significantly higher attack rate on *P. virginalis*. 

Based on these results, we can conclude that crayfish populations, including native species (e.g., genus *Astacus* and *Austropotamobius* for European regions), in freshwater ecosystems may be exposed to predation stress by *N. melanostomus* similar to that on *A. aquaticus*. Lawton et al. [83] reported that the predator attack rate decreases and handling time is elevated when alternative prey items are available [83], and Colton [53] demonstrated that, in a multi-prey system, both handling time and attack rate vary with quantity and characteristics of the second most available prey item [53]. However, our experimental design did not allow analysis of those parameters with respect to prey species separately in the multiple-prey exposures. Handling time and attack rate in our multi-prey trials reached values different from those that would be expected in single-prey exposures. Regardless of the proportion of *P. virginalis*, the prey item considered to be a driver of the *N. melanostomus* attack rate, on the overall offered prey amount, *N. melanostomus* showed a higher attack rate in both 3:1 ratios compared to 1:1 or *A. aquaticus* offered separately. At 1:1, the attack rate was similar to that in the system with only *A. aquaticus*. In both 3:1 trials at lower densities, the attack rate was positively affected, while, at higher densities, *N. melanostomus* handling time was prolonged compared to expectations based on results gained in the single-prey systems, implying ongoing predator switch to the alternative prey. The prey alternation could be more challenging when prey species occur in unequal quantities. This is in agreement with Colton [53], who stated that the addition of a prey species to a system leads to additional interactions and behaviour changes, and the food system becomes unpredictable. Lawton et al. [83] reported reduced predator pressure on individual prey in such conditions due to the increased handling time and depressed attack rate. However, our data clearly showed that addition of a second prey item led to an increase in *N. melanostomus* attack rate as well as elevated impact on the prey community. In addition, our study confirms the value of multi-species experimental design in ecological studies to gain a more realistic assessment of predator impact upon prey communities. 

Several studies have documented *N. melanostomus* feed selectivity [30,84,85] that differs with locality. The optimal foraging theory states that a predator will maximize energy profit to cost with respect to prey acquisition and processing [86]. Prey selectivity in aquatic ecosystems is affected by multiple factors including prey availability [87]; predator experience [56]; prey size, morphology, and colour [87,88]; and water turbidity [89,90]. The latter is demonstrated by *N. melanostomus* diet shift to easily available prey under experimental conditions of high turbidity [89]. Therefore, it can be assumed that prey exhibiting an effective escape response and/or high mobility will be less preferred by predators [86]. However, studies of *N. melanostomus* feed selectivity have often shown contradictory results, with respect to preferences for native [91,92] or non-native [93] species. In addition, overexploitation of certain benthic species regardless of abundance has been observed [30,84,85] and confirmed by our findings of no species-differences in the number of prey killed when presented in equal numbers, while at 3:1, *N. melanostomus* killed significantly more specimens of the prevalent species. These findings support the hypothesis that *N. melanostomus* often shows indiscriminate foraging, taking the most readily available prey and easily switching to another source [30,94]. The ineffectiveness of crayfish tail-flip escape strategy against *N. melanostomus* predation was also shown. An exception was 1:1 presentation of prey at density of 100 ind/box, when *N. melanostomus* killed significantly more *P. virginalis* than *A. aquaticus*, possibly showing predator food preference after satiation [45]. 

Although the focus is generally on the direct consumption of prey, this is not the only means by which predator ecological impact may occur [95]. We observed that non-consumptive mortality (NCM) may have an even higher effect on prey populations than direct predation [96]. This component of predator behaviour, also known as waste or surplus killing, has been observed in invertebrates [63,97,98] and mammals [99,100,101]. Ignoring NCM may cause a significant underestimation of predator ecological impact [102] as well as energy transfer among trophic levels [97]. In our experimental exposures, *N. melanostomus* exhibited a high rate of NCM, indicating its potential role in the effect of this predator on prey population abundance and ecosystem function. Our observed NCM is in contrast with previous studies of *N. melanostomus* FR with fish larvae as prey [52,77] in which no NCM was observed. In mammals, NCM is usually connected either with an ineffective anti-predator response due to lack of co-evolution with the predator [101] or to lack of prey escape response as a consequence of isolated short-term events [102]. In invertebrates, it seems that the satiation level determines whether the prey is consumed. However, hunting and killing of prey are probably directed by mechanisms [97] in invertebrates that differ from that of vertebrates [103]. Johnson et al. [97] assumed that an empty midgut may stimulate predatory damselfly nymphs to capture more prey than can be processed due to filled foregut. It seems that an effect of satiation was not confirmed in our experiment, since *N. melanostomus* killed both prey species without their consumption after 24 h starvation, even at the lowest densities. Although the NCM has usually been reported to increase as prey density rises [63,97,98], we did not find a correlation of NCM rate and prey density in *N. melanostomus*, and the proportion of NCM in total prey mortality ranged from 0–100% (33.4 ± 39.2%). 

Fantinou et al. [63] described NCM elevation at temperatures outside the predator thermal optimum, i.e., in stressful conditions. Similarly, Veselý et al. [98] in a study of *Aeschna cyanea* nymphs, and Jedrzejewska and Jederzejewski [100] in *Mustela nivalis*, described higher NCM at lower temperature. However, it is unclear whether the low temperature directly caused change of predator behaviour or influenced prey occurrence and/or behaviour and subsequently predator response. In our study, the temperature ranged within the optimum range reported for *N. melanostomus* [104]. We can assume that a potential reason for observed high NCM values might be the absence of shelter as a possible trigger of stress, although we have no evidence supporting this assumption or quantifying its importance in the wild in *N. melanostomus*. 

*Neogobius melanostomus* successfully exploited both hard-bodied prey species differing in escape strategy without showing a distinct preference. The simultaneous effects of high *N. melanostomus* foraging efficiency on *P. virginalis* and previously documented successful competition of *N. melanostomus* for shelter with crayfish [105] may demonstrate a potential to regulate *P. virginalis* populations in the wild. Bovy et al. [106] pointed out that a destabilization effect of predator presence on prey populations is negatively correlated with prey reproduction and dispersal abilities. Therefore, despite a strong interaction between *P. virginalis* and *N. melanostomus* as invasive non-native species, an eradication effect is less likely in established *P. virginalis* populations due to its high fertility rate and overall reproduction ability [107]. However, for native crustaceans, including indigenous European crayfishes that are threatened for many reasons [7,62] and exhibit lower fecundity [108], *N. melanostomus* may pose a serious risk. Particularly with regards to increasing records of *N. melanostomus* in smaller tributaries [30,109,110] inhabited by native crayfish, this can be crucial for continuing crayfish existence. More attention should be focused on identifying and clarifying non-consumptive mortality in the wild as a potential element of *N. melanostomus* foraging behaviour. The reason for ineffective predation in *N. melanostomus* is unclear, and this is one of the first laboratory foraging studies to report non-consumptive predation in fish. Both indiscriminate foraging behaviour and non-consumptive mortality are important factors that should be taken into consideration for quantification of *N. melanostomus* impact on native crustaceans in freshwater ecosystems.

## 5. Conclusions

Although *N. melanostomus* shows comparable predation pressure on both preys, it can be a threat to the population stability of already endangered crustaceans such as crayfish. Effective control to limit further spreading of *N. melanostomus* to tributaries should be a priority. There is a need for more multiple-prey studies, as quantification of *N. melanostomus* impact on the macrozoobenthic community based on the single prey model may be insufficient. In addition to prey species, their density and relative proportions can significantly influence the *N. melanostomus* foraging efficiency.

## Figures and Tables

**Table 1:** Linear coefficient P_1_ of logistic regression of *Neogobius melanostomus* relative to prey species and stocking ratio.

Exposure	Linear Coefficient P_1_	SE	*p*-Value
*A. aquaticus*	−1.145	0.364	0.002
*P. virginalis*	−1.107	0.360	0.002
1:1 *A. aquaticus/P. virginalis*	−1.047	0.360	0.004
3:1 for *A. aquaticus*	−1.580	0.365	<10^−4^
3:1 for *P. virginalis*	−1.302	0.361	<10^−3^

**Table 2:** Confidence intervals (95% CI) of handling time and attack rate of *Neogobius melanostomus* relative to prey species and presentation (separately or mixed). In multiple prey trials, *Asellus aquaticus* and *Procambarus virginalis* were offered at ratios of 1:1, 3:1 or 1:3.

Parameter	Prey	Lower Limit of 95% CI	Mean	Upper Limit of 95% CI	*p*-Value
Attack rate	*A. aquaticus*	2.573	3.094	3.615	<10^−6^
	*P. virginalis*	4.433	5.640	6.848	<10^−6^
	1:1 *A. aquaticus*/*P. virginalis*	2.830	3.568	4.307	<10^−6^
	3:1 for *A. aquaticus*	5.900	7.724	9.548	<10^−6^
	3:1 for *P. virginalis*	4.491	5.825	7.158	<10^−6^
Handling time	*A. aquaticus*	0.008	0.010	0.012	<10^−6^
	*P. virginalis*	0.011	0.012	0.014	<10^−6^
	1:1 *A. aquaticus*/*P. virginalis*	0.011	0.013	0.015	<10^−6^
	3:1 for *A. aquaticus*	0.016	0.018	0.019	<10^−6^
	3:1 for *P. virginalis*	0.014	0.016	0.018	<10^−6^

## Data Availability

The data presented in this study are available from the corresponding author upon request.
